# Combined Treatments Reduce Chilling Injury and Maintain Fruit Quality in Avocado Fruit during Cold Quarantine

**DOI:** 10.1371/journal.pone.0140522

**Published:** 2015-10-26

**Authors:** Velu Sivankalyani, Oleg Feygenberg, Dalia Maorer, Merav Zaaroor, Elazar Fallik, Noam Alkan

**Affiliations:** 1 Department of Postharvest Science of Fresh Produce, Volcani Center, Agricultural Research Organization, Bet Dagan, Israel; 2 Robert H Smith, Faculty of Agriculture, Food and Environment, The Hebrew University of Jerusalem, Rehovot, Israel; University of Campinas, BRAZIL

## Abstract

Quarantine treatment enables export of avocado fruit (*Persea americana*) to parts of the world that enforce quarantine against fruit fly. The recommended cold-based quarantine treatment (storage at 1.1°C for 14 days) was studied with two commercial avocado cultivars ‘Hass’ and ‘Ettinger’ for 2 years. Chilling injuries (CIs) are prevalent in the avocado fruit after cold-quarantine treatment. Hence, we examined the effect of integrating several treatments: modified atmosphere (MA; fruit covered with perforated polyethylene bags), methyl jasmonate (MJ; fruit dipped in 2.5 μM MJ for Hass or 10 μM MJ for Ettinger for 30 s), 1-methylcyclopropene (1-MCP; fruit treated with 300 ppb 1-MCP for 18 h) and low-temperature conditioning (LTC; a gradual decrease in temperature over 3 days) on CI reduction during cold quarantine. Avocado fruit stored at 1°C suffered from severe CI, lipid peroxidation, and increased expression of chilling-responsive genes of fruit peel. The combined therapeutic treatments alleviated CI in cold-quarantined fruit to the level in fruit stored at commercial temperature (5°C). A successful therapeutic treatment was developed to protect ‘Hass’ and ‘Ettinger’ avocado fruit during cold quarantine against fruit fly, while maintaining fruit quality. Subsequently, treated fruit stored at 1°C had a longer shelf life and less decay than the fruit stored at 5°C. This therapeutic treatment could potentially enable the export of avocado fruit to all quarantine-enforcing countries. Similar methods might be applicable to other types of fruit that require cold quarantine.

## Introduction

With the increase in global trading of agricultural produce, there is a rising danger of invasion of insect species into new areas, where they are considered quarantine-required pests [1]. The Mediterranean fruit fly *Ceratitis capitata* (Wiedemann) (Diptera: Tephritidae) is one such quarantine-requiring insect (as defined by the OEPP/EPPO) that is prevalent in Asia, Africa, southern and mid America and Mediterranean countries. Many countries that do not have these pests enforce strict quarantine measures [2], thus preventing the export of many fruits, avocado among them. To enable the export of fresh agricultural produce to those countries, it is essential to develop a protocol that will kill all insects without compromising fruit quality. There are several known post-harvest quarantine treatments that are applied before or during export, such as heat, cold-temperature or radiation treatments [3, 4]. All of these treatments have limitations: radiation treatments are expensive, heat treatments often impair fruit quality, and cold treatments are usually accompanied by chilling injuries (CIs).

Cold-quarantine treatment, consisting of 14–18 d at 1.1–2.2°C has proven efficient at killing the Mediterranean fruit fly in various fruits [5]. This treatment was approved by the USDA as a mandatory cold-based quarantine treatment for 21 different fruit, including avocado [6]. The recommended optimum cold storage temperature for avocado varies between 5 and 10°C based on cultivar, maturity and harvest season. The avocado cultivars Ettinger and Hass are stored commercially at 5°C, storage below this temperature causes CI [7]. Chilling stress alters membrane fluidity by oxidative stress, which induce peroxidation and breakdown of lipids in the membrane [8]. Chilling also causes plant metabolic changes by activation of signal transduction that regulates stress responsive genes as: lipoxygenase (LOX), fatty-acid desaturase (FAD) and heat shock protein (HSP) to cope with stress [9, 10]. Many studies have focused on conditioning fruit to increase their resistance to low temperatures in order to extend their storage period. For example, modified atmosphere (MA) has been shown to significantly reduce CI in mango avocado and coconut [11–13], and significantly extend avocado storage life [14]. Creating a MA for mango fruit reduces water loss and thus decreases CI [11, 14]. Wax coating of grapefruit and pomegranate has also been found to significantly reduce the progress of CI symptoms [15, 16]. Application of the defense hormone methyl jasmonate (MJ) reduces the rate of CIs in mango, avocado, pepper and pomegranate fruit [17–19]. Heat treatment before storage increases membrane integrity and decreases ion leakage, and ultimately reduces CI in tomato fruit [20]. Similarly, heat treatment reduced CIs in mango, avocado and pomegranate fruit [21–23]. Therefore, various heat treatments have been suggested to prevent damage caused by cold-quarantine treatments in avocado fruit [24–26]. Indeed, those heat treatments reduced external CIs in avocado, but they did not eliminate them. Interestingly, low-temperature conditioning (LTC) was more effective at reducing external CI of ‘Hass’ avocados and addition of a heat treatment did not show an additive effect [27]. Hence, to prevent damage incurred during cold quarantine in avocado, we integrated LTC with other treatments, namely MA and MJ. Plant hormone MJ, modified atmosphere and low temperature conditioning have been shown to regulate stress responsive genes LOX, FAD and HSP to alleviate chilling injuries in fruits and vegetables [10, 28–32]. We found that the combined treatments increased avocado resistance to suboptimal temperature storage during the cold-quarantine treatment, thereby preventing CI and maintaining fruit quality.

## Materials and Methods

### Plant Material

Mid-season avocado fruit (*Persea americana* cultivars Ettinger and Hass) were harvested in the month of January (Hass) and October-November (Ettinger) in 2013 and 2014. Fruit were obtained from Granot storage house (Israel) 2–3 h after harvest and transported (less than an hour) without bruising to the Agricultural Research Organization, Israel. Uniform, unblemished fruit weighing ~250 g for ‘Ettinger’ and 200 g for ‘Hass’, with 14 and 20 fruit per 4 kg carton box, respectively, were used for the experiments. The experiments were repeated twice for each cultivar. In all experiments, fruit pulp dry weight was 22–26% and considered to be mature enough to ripen.

### Therapeutic treatments and suboptimal temperature storage

To evaluate the effect of therapeutic treatments on reducing CI in avocado fruit while maintaining its quality, nine sets of treatments were carried out. Fruits were treated with: (1) methyl jasmonate (MJ)–fruits were dipped in 2.5 μM (Hass) or 10 μM (Ettinger) MJ for 30 s; (2) modified atmosphere (MA)–fruit were covered with a perforated (30 holes of 0.5 mm), low-density (40 μm) polyethylene bags (StePac, Israel; bags were left open on the first day of covering to avoid high humidity). The relative humidity inside the bag was about 97–8% with no condensation, CO_2_ concentration inside the bag was about 3–5% and O_2_ concentration was about 13–15%; (3) LTC–a gradual reduction in temperature over 3 days—12°C (RH 62%) on day 1, 5°C (RH 50%) on day 2 and then storage at 1°C (RH 45%)); (4–9) combinations of treatments 1–3. The negative control was non-treated fruit stored at 5°C for 3 weeks (with a fruit core temperature of 5 ± 0.3°C, data not shown). The positive control was non-treated fruit stored at 1°C for 15 days (with a fruit core temperature of 1 ± 0.25°C, S1 Fig), followed by storage at 5°C for the rest of the 3 weeks of cold storage. Cold storage was followed by 7 to 10 days of shelf storage at 20°C (RH 64%).

Another experiment was conducted with ‘Hass’ to optimize the LTC and to determine the effect of methylcyclopropene (1-MCP). Fruits were treated with: (1) LTC 3d –a gradual reduction in temperature over 3 days—day 1 at 12°C, day 2 at 5°C, followed by storage at 1°C; (2) LTC 5d –a gradual reduction in temperature over 5 days, at 12, 9, 6, 3°C for 1 day each, followed by storage at 1°C; (3) treatment with 300 ppb 1-MCP (RIMI, Israel) at 20°C for 18 h; (4) combinations of treatments 1–3.

Four carton boxes, each with 14 fruits (Ettinger) or 20 fruits (Hass), were used per treatment in each experiment. The temperature in the cold-storage room was monitored by a DAQ tool—Double Strand wire logger/Data Acquisition control system (T.M.I Barak ltd, Israel). Fruit core temperature was monitored for the non-treated fruit and the combined treatments using a MicroLite data Logger LITE5032P-EXT-A (Fourier technologies, Israel), by inserting the probe on near calyx part of fruit in 5cm deep (S1 Fig).

### Analysis of avocado fruit response to cold-quarantine treatments

CI symptoms in the treated and non-treated ‘Hass’ and ‘Ettinger’ avocado were determined by external appearance of the fruit after cold storage (5°C or 1°C for 15 days, followed by storage at 5°C for the rest of the 3 weeks of cold storage) and after another 7 days of shelf storage at 20°C. Severity of external CIs such as blackening and pitting of the skin surface in the treated and non-treated ‘Ettinger’ and ‘Hass’ fruit were evaluated and rated on a relative scale of 0 to 3 (CI index) where 0 = no chilling injuries; 1 = up to 25% surface area showing CI on skin surface of fruit; 2 = 26–50% CI, and 3 = 50–100% of skin surface showing CI. Other physiological parameters were checked and presented as percentage of fruit showing decay, percentage of fruit showing blossom-end rot and firmness in Newton (N). Fruit firmness was measured using an electronic penetrometer (LT-Lutron FG-20 kg, Indonesia) with an 11-mm probe at two points on the equatorial line of each fruit (20 measurement/treatment) via peel. Since ‘Hass’ avocado tends to change color from dark green to dark gray at the ripening stage, change in fruit color (hue) was measured among the treated and non-treated ‘Hass’ avocado cultivars using a chromometer (Minolta, LR-400/410) at two points on the equatorial line of each fruit (20 measurement/treatment). Internal browning and seed browning were evaluated after shelf storage (20 measurement/treatment).

### Evaluation of lipid peroxidation after cold storage by *In Vivo* imaging system (IVIS)

‘Hass’ avocado fruit were randomly selected after 15 days of cold storage at 5°C or 1°C or 1°C with treatment (MJ, MA and LTC) to detect lipid peroxidation level using a Pre-clinical *In Vivo* Imaging Systems (IVIS, PerkinElmer, USA). Fruit were kept in the dark for 1–2 h prior to the evaluation. Lipid peroxidation in the fruit was detected and visualized by autoluminescence of peroxide lipids as described previously [33, 34], using a programmed sequence of autoluminescence for 20 min with emission at 640–770 nm and excitation-block, a binning factor of 8, and an F-stop factor of 1. Lens XFOV-24 was used for focus in the field of view E. The auto-luminescence was recorded by highly sensitive charge-coupled-device (CCD) camera. The optical luminescent image data was displayed in pseudocolor that represents intensity in terms of Radiance (photons/sec/cm^2^/steradian). The measurements were repeated three times with different fruits.

### Determination of relative expression of chilling-responsive genes by quantitative real-time PCR (qRT-PCR)

Peel tissue was collected from the positive and negative ‘Hass’ avocado fruit controls, and from the combined treatments (MA, MJ, and LTC) during 18 days of cold storage. Total RNA was extracted from the peel tissue as described by Djami-Tchatchou and Straker [35]. Total RNA was treated with DNase (TURBO DNA-free Kit, Ambion Life Technologies, USA) according to the manufacturer's instructions. Total RNA (1 μg) was used for cDNA construction using the RevertAid First-Strand cDNA Synthesis kit (Thermo Scientific, USA) according to the manufacturer's instructions. cDNA samples were diluted 1:10 to the final template concentration and used for qRT-PCR. The relative expression of cold-induced genes encoding omega-3-fatty acid desaturase 7 (*FAD7*; FD505476.1), heat-shock protein (*HSP II 17*.*6*; CK748339.1) and lipoxygenase (*LOX*; FD505476.1) was evaluated by qRT-PCR analysis conducted with a Step One Plus Real-Time PCR (Applied Biosystems, USA). PCR amplification was performed with 3.4 μl of diluted cDNA template in 10 μl reaction mixture containing 5 μl Sybr Green (Applied Biosystems) and 300 nM primers. qRT-PCR analysis was conducted with the corresponding primer sets of the selected genes: forward, 5'-CACAGGACGCATCACCAGAA-3' and reverse, 5'-TGCGGGAAACATCATCCAA-3' for *FAD7*, forward, 5'-AGGCGATGGCGTCAACTC-3' and reverse, 5'-CCTCTCGCCGCTAATTACCA-3' for *HSP II 17*.*6*, forward, 5'-AAGGCTCGGTGGTGTTGATG-3' and reverse, 5'-TCGCCATGTTCTGCACTGA-3' for *LOX*, and forward, 5'- AGCTCGCTTATGTGGCTCTTGACT-3' and reverse, 5'- TCTCATGGATTCCAGCAGCTTCCA-3' for the housekeeping *actin* gene. PCR was carried out using the following cycling program: 10 min at 94^°^C, followed by 40 cycles of 94^°^C for 10 s, 60^°^C for 15 s, and 72^°^C for 20 s. The expression of the selected genes was normalized to that of *actin* and the relative expression was calculated using relative standard curve with Step One software v2.2.2 (Applied Biosystems). Each treatment at each time point consisted of three biological repeats and two technical replicates.

### Statistical analysis

Data were analyzed by one-way analysis of variance (ANOVA) and differences were compared by Duncan's multiple-range test using Sigma Stat 3.5 software (Systat Software Inc., USA). *P <* 0.05 was considered statistically significant.

## Results

### The protective effect of therapeutic treatments on ‘Ettinger’ and ‘Hass’ avocado during cold-quarantine treatment

Mid-season ‘Ettinger’ and ‘Hass’ avocado fruit were stored at 5°C or 1°C for 15 days, followed by storage at 5°C for the rest of the overall 3 weeks in cold storage. The cold storage was followed by 7 to 10 days of shelf storage at 20°C. The protective effect of the therapeutic treatments was tested on cold-quarantined ‘Ettinger’ and ‘Hass’ fruit. The fruit were treated with MA, MJ or LTC, or their combinations, prior to the cold-quarantine treatment. Non-treated fruit stored at 5°C or 1°C served as negative and positive controls, respectively. The experiment was carried out in two seasons (2013 and 2014) for both ‘Ettinger’ and ‘Hass’. Representative results of one characteristic experiment for each cultivar are presented in the figures.

### Combined therapeutic treatments minimize CI in ‘Ettinger’ and ‘Hass’

**Fig 1:**
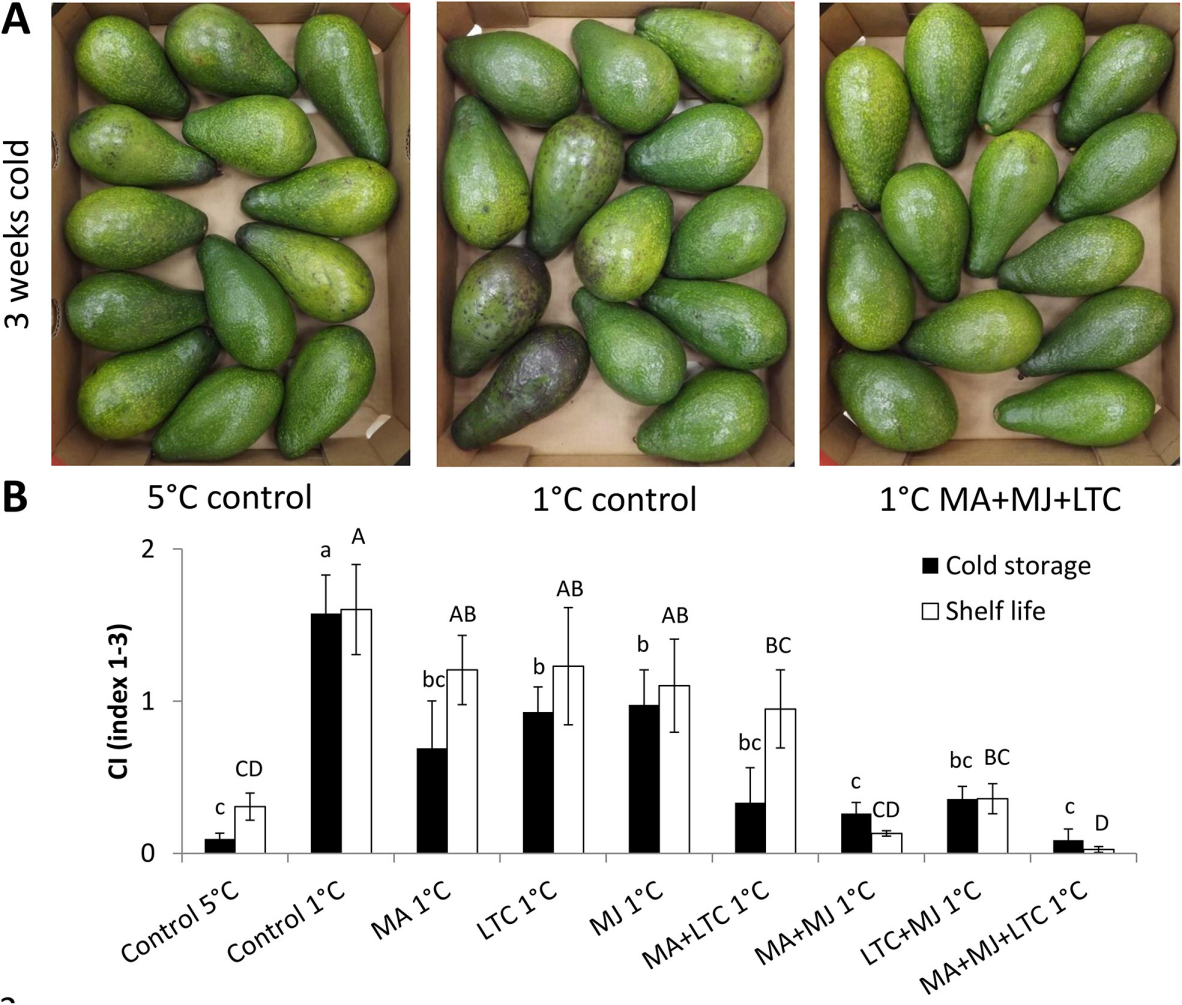
Chilling symptoms of ‘Ettinger’ after cold-quarantine treatments. Evaluation of chilling-injury (CI) symptoms in treated [modified atmosphere (MA), methyl jasmonate (MJ), low-temperature conditioning (LTC)] and non-treated ‘Ettinger’ after cold-quarantine treatments (15 days at 5°C or at 1°C, then storage at 5°C for the rest of the overall 3 weeks in cold storage), followed by 7 days of shelf storage. (A) Representative pictures of ‘Ettinger’ fruit after 3 weeks of cold storage followed by shelf storage. (B) CI severity ranked from 1–3 after cold storage (black column) and further shelf storage (white column). Data are mean ± SE. Different letters (lowercase letters and uppercase letters refer to after cold storage and shelf life, respectively) indicate significant differences at *P* < 0.05 by one-way ANOVA and Duncan's multiple range test.

**Fig 2:**
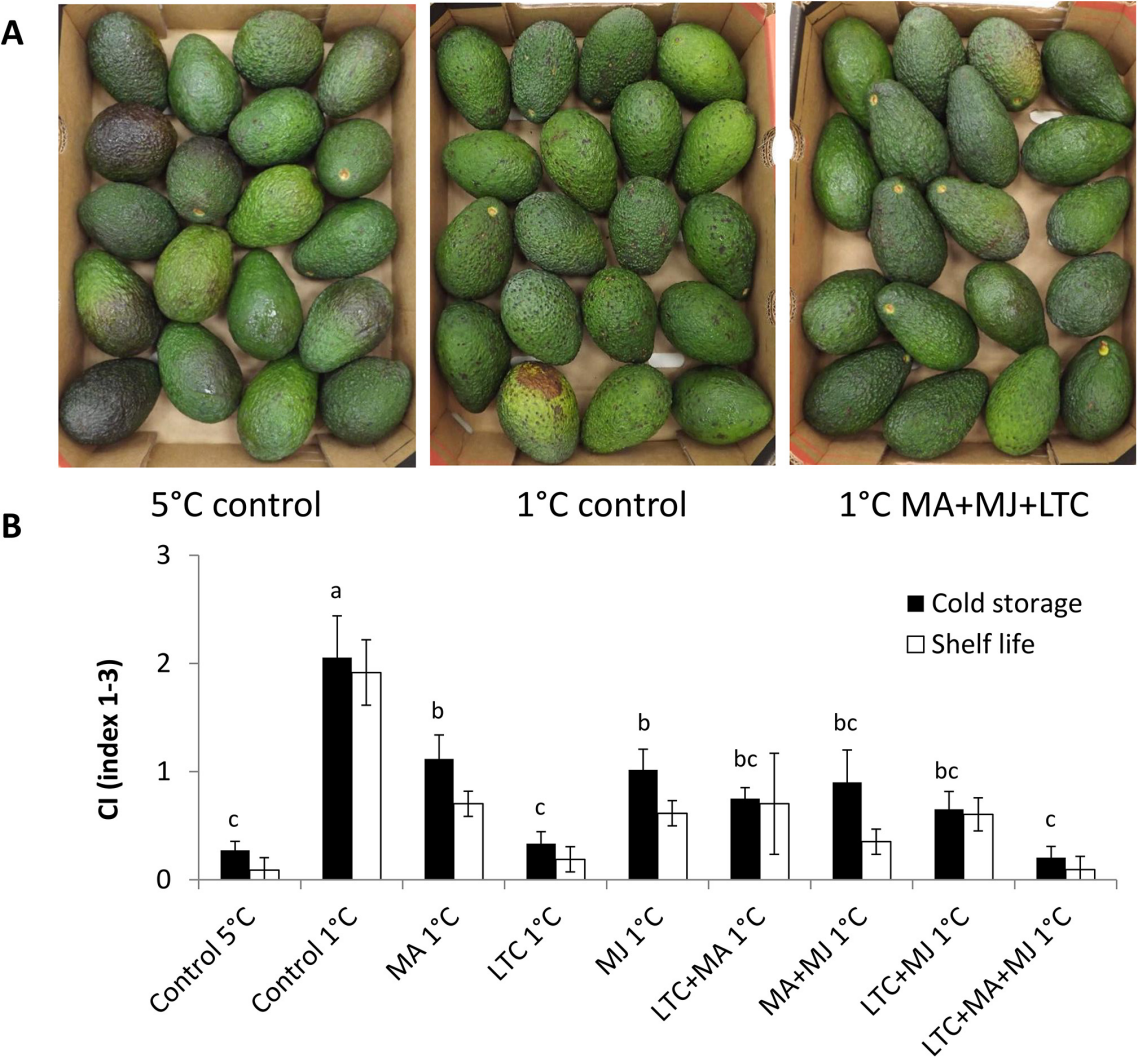
Chilling symptoms of ‘Hass’ fruit during cold-quarantine treatments. Evaluation of chilling-injury (CI) symptoms in ‘Hass’ during cold-quarantine treatments (15 days at 5°C or at 1°C, then storage at 5°C for the rest of the overall 3 weeks of cold storage), followed by 7 days of shelf storage. (A) Representative pictures of ‘Hass’ fruit after 3 weeks of cold storage followed by shelf storage. (B) CI severity ranked from 1–3 after cold storage (black column) and further shelf storage (white column). Data are mean ± SE. Different letters indicate significant differences at *P* < 0.05 by one-way ANOVA and Duncan's multiple range test.

As expected, both ‘Ettinger’ (Fig 1A) and ‘Hass’ (Fig 2A) fruit stored at 5°C (negative control) showed barely any CI symptoms, whereas fruit stored at 1°C (positive control) showed severe CI symptoms such as black spots and pitting. Therapeutic treatments with MA, MJ or LTC had a pronounced protective effect on fruit during suboptimal-temperature storage and reduced the severity of the CI when applied individually (Figs 1B and 2B). The combination of two therapeutic treatments enhanced the reduction in CI severity compared to either treatment alone. The combination of all three therapeutic treatments (MA, MJ and LTC) had the greatest protective effect on fruit and minimized CI to the level of negative control fruits stored at 5°C (Figs 1 and 2). Storage at lower temperatures delays ripening, and therefore fruit stored at 1°C ripened slowly, reaching full ripening after 6–7 days of shelf storage. Therefore, fruit subjected to the combined treatment showed almost no decay even after 10 days of shelf storage, whereas commercially stored fruit (5°C) started to decay and reached a non-marketable stage (S3 Fig).

### Therapeutic treatment effects on ‘Ettinger’ and ‘Hass’ fruit physiology

**Fig 3:**
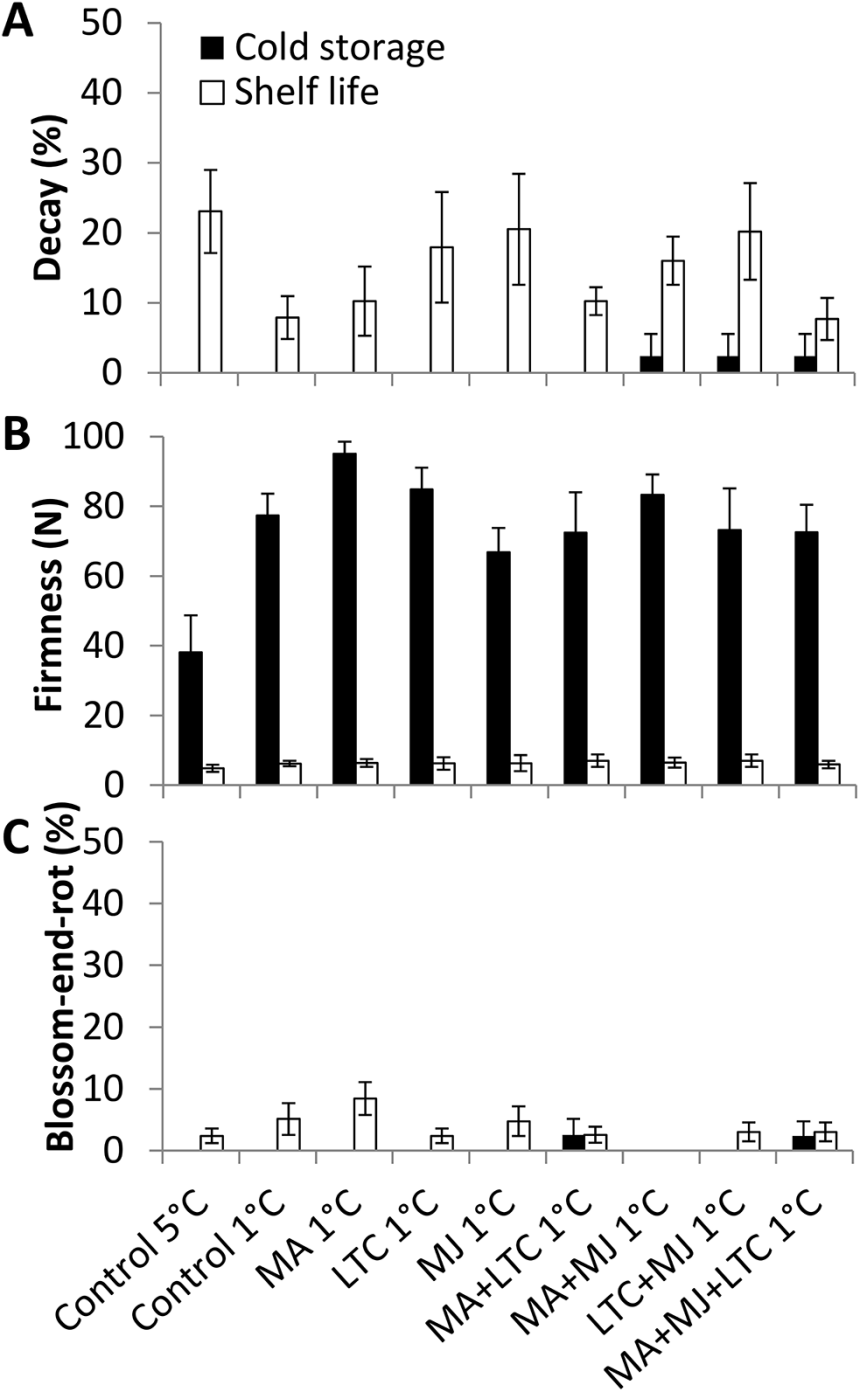
Evaluation of physiological parameters of ‘Ettinger’ fruit during cold-quarantine treatments. Physiological parameters of treated [modified atmosphere (MA), methyl jasmonate (MJ), low-temperature conditioning (LTC)] or non-treated ‘Ettinger’ after cold storage (black column) and further shelf storage (white column). (A) Overall decay displayed in percentage. (B) Firmness displayed in Newton. (C) Blossom-end rot displayed in percentage. Data are mean ± SE.

**Fig 4:**
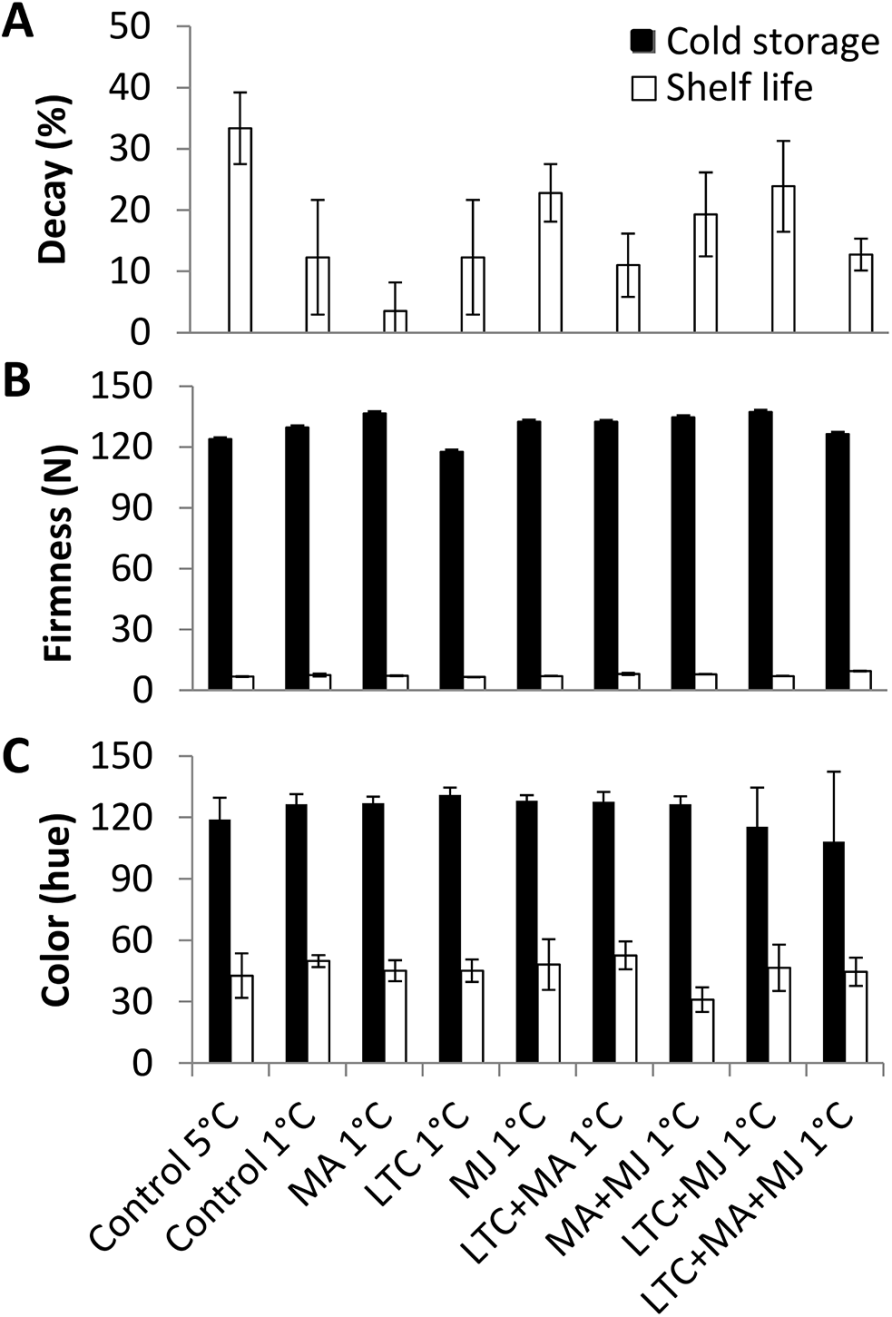
Evaluation of physiological parameters of ‘Hass’ fruit during cold-quarantine treatments. Physiological parameters of treated [modified atmosphere (MA), methyl jasmonate (MJ), low-temperature conditioning (LTC)] or non-treated ‘Hass’ after cold storage and further shelf storage. (A) Overall decay displayed in percentage. (B) Firmness displayed in Newton. (C) Fruit color displayed in hue. Data are mean ± SE.

Fungal-derived decay appeared mainly after shelf-life storage (Figs 3A and 4A). Fruit stored at 1°C had less decay overall. The combined treatments significantly reduced total decay in both ‘Ettinger’ and ‘Hass’ (Figs 3A and 4A). In ‘Hass’, the LTC treatment significantly reduced total decay (Fig 4A) and CI (Fig 2B). Therefore, the combined treatments and the LTC treatment were the best therapeutic treatments for ‘Hass’ avocado fruit.

Suboptimal temperature storage at 1°C with or without therapeutic treatments had an effect on ‘Ettinger’ but not ‘Hass’ fruit firmness (Figs 3B and 4B). However, suboptimal temperature storage (1°C) did not influence blossom-end browning in ‘Ettinger’ (Fig 3C) or color change in ‘Hass’ (Fig 4C). In addition, inner browning was barely detected with any of the treatments in either ‘Ettinger’ or ‘Hass’ (see representative pictures in S2 Fig).

### Effect of 1-MCP therapeutic treatment in ‘Hass’

**Fig 5:**
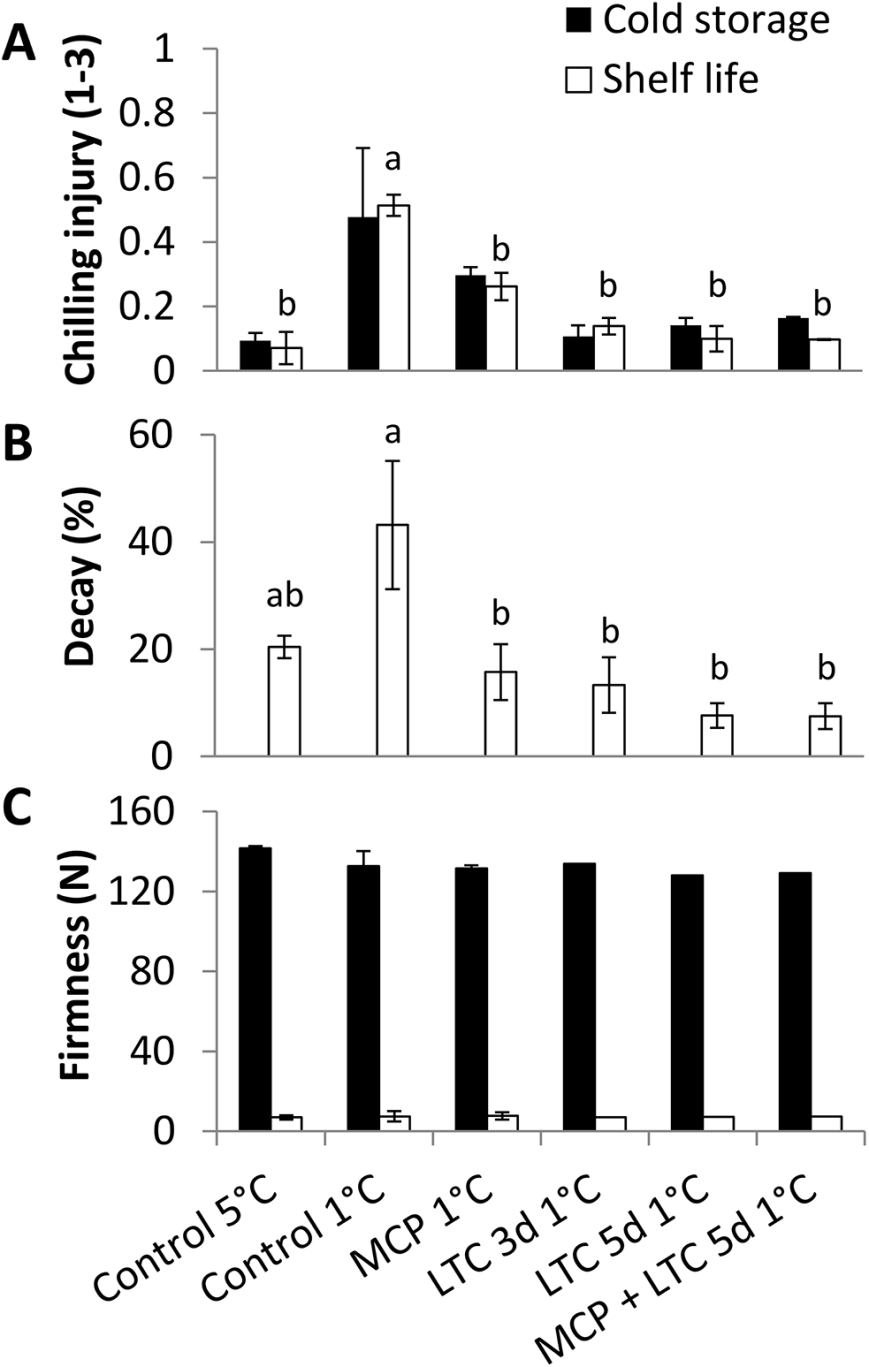
Evaluation of physiological parameters of ‘Hass’ fruit during cold-quarantine treatments. Treated (LTC 3d or 5d: 3 or 5 days of low-temperature conditioning, MCP: 1-MCP 150 ppm) or non-treated ‘Hass’ fruit were checked for physiological parameters after cold storage (15 days at 5°C or at 1°C, then storage at 5°C for the rest of the overall 3 weeks in cold storage), followed by 7 days of shelf storage at 20°C. (A) Chilling injuries (CI index 1–3). (B) Overall decay displayed in percentage. (C) Firmness displayed in Newton. Data are mean ± SE. Different letters indicate significant difference at *P* < 0.05 by one-way ANOVA and Duncan's multiple range test.

1-MCP is known to delay CI [36] and is used commercially to delay ripening. LTC treatment showed a promising effect on CI reduction in ‘Hass’. We further examined the protective effect of 1-MCP + LTC on cold-quarantined ‘Hass’. Indeed, a minor reduction in total decay and CI was observed after 1-MCP treatment (Fig 5A and 5B). LTC treatment had a better protective effect against CI and decay than the 1-MCP treatment (Fig 5A and 5B). The gradual LTC 5d and the short LTC 3d had a similar effect on CI reduction (Fig 5). No additive effect was found for the combination of 1-MCP and LTC (Fig 5A and 5B). None of the tested treatments had any influence on fruit firmness (Fig 5C).

### Therapeutic treatment influence on expression of chilling-responsive genes

**Fig 6:**
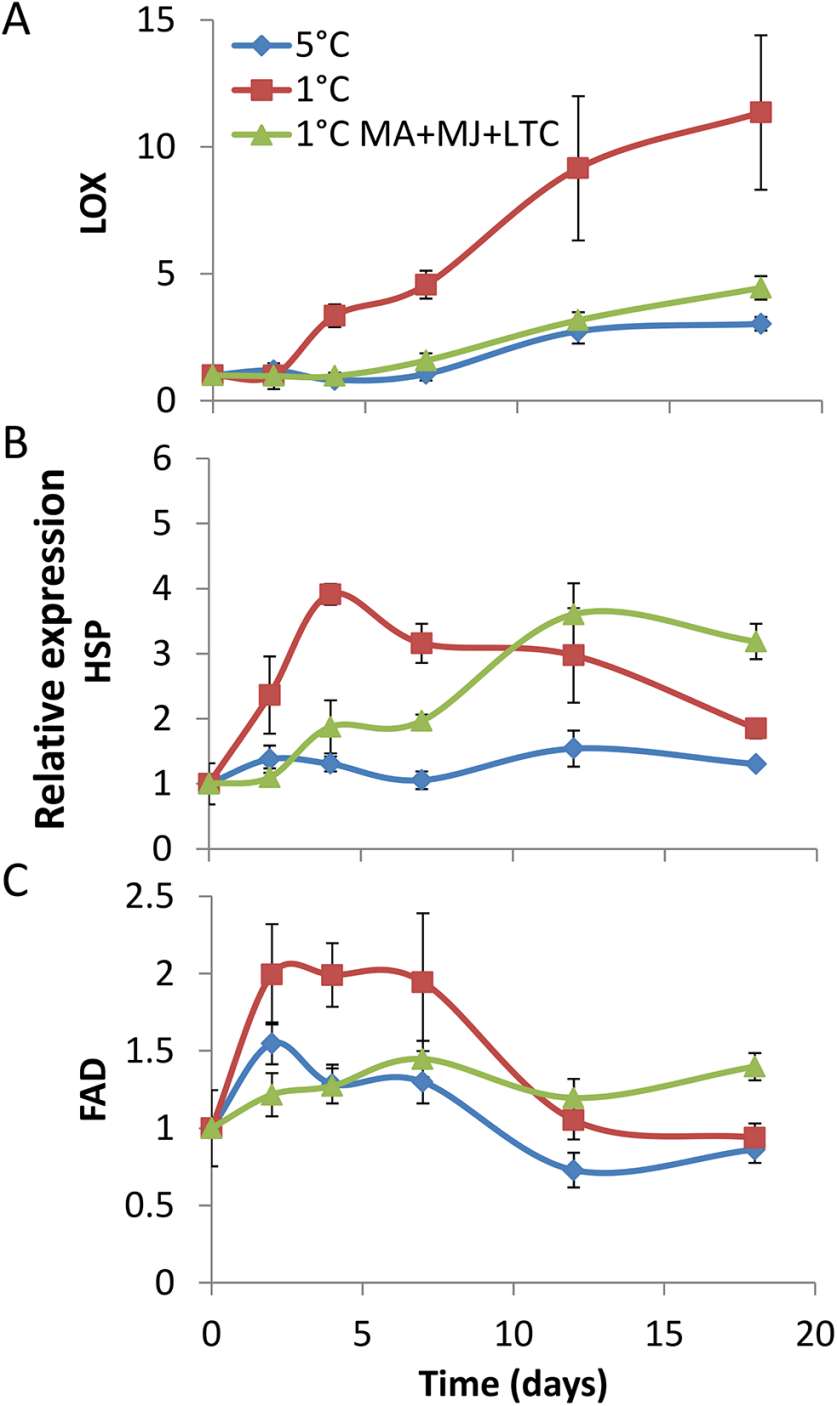
Relative expression of genes related to cold-response. Relative expression of genes in peel tissue of ‘Hass’ during 18 days of cold storage at 5°C or at 1°C with or without the combined treatments [modified atmosphere (MA), methyl jasmonate (MJ), low-temperature conditioning (LTC)] encoding: (A) lipoxygenase (LOX), (B) heat-shock protein (HSP), (C) fatty acid desaturase (FAD).

To better understand the effect of the therapeutic treatments on chilling response of avocado, relative expression of the chilling-responsive genes: Lipoxygenase (*LOX*), Fatty acid desaturase (*FAD*), and heat shock protein (*HSP*) in the peel tissue of ‘Hass’ were examined by qRT-PCR. The relative expression level of *LOX* increased gradually over 3 weeks of suboptimal temperature storage at 1°C more than threefold compared to fruit stored at 5°C (Fig 6A). Interestingly, fruit receiving combined treatments (MA, MJ and LTC) followed by storage at 1°C showed lower expression levels, similar to fruit stored at 5°C (Fig 6A). The relative expression of *HSP* and *FAD* increased rapidly in fruit stored at 1°C compared to fruit stored at 5°C, and started to decline on days 4 and 8, respectively (Fig 6B and 6C). Interestingly, *HSP* and *FAD* expression gradually increased in the combined treatments, reaching a maximum after 18 days of suboptimal temperature storage.

### Combined therapeutic treatments minimize lipid peroxidation in avocado during cold storage

**Fig 7:**
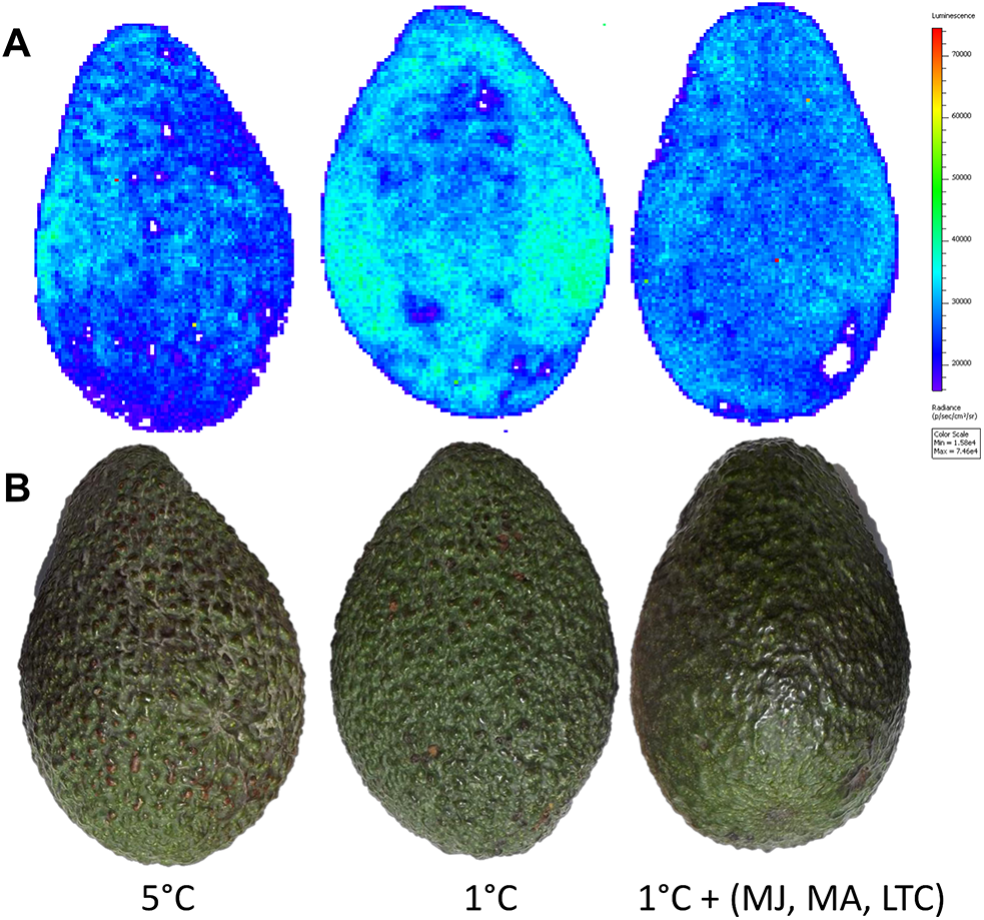
Luminescence and light image of ‘Hass’ avocado fruit in response to cold storage. Luminescence and light image were captured and visualized after 15 days of cold storage at 5°C or 1°C or 1°C with the combined treatments (MJ, MA, LTC). (A) Luminescence (20 min of autoluminescence, emission: 647–770 nm) indicating lipid peroxidation. (B) Light image.

Lipid peroxidation is known to be a major indicator of CI in various plants [37, 38], and its fluorescence has been observed in avocado fruit [39]. Lipid oxidation can also be detected by spontaneous photon luminescence [34]. Lipids have been found to emit persistent autoluminescence after oxidation and emit light at wavelengths higher than 600 nm [33]. We evaluated luminescence of the peroxidized lipid in ‘Hass’ avocado fruit after 15 days of cold storage at 1°C (positive control) or 5°C (negative control) or 1°C with combined therapeutic treatment (MJ, MA and LTC) using IVIS. The autoluminescence was significantly higher in fruit stored at 1°C compared to those stored at 5°C and 1°C with combined therapeutic treatment (Fig 7A).

## Discussion

A good quarantine treatment must kill all pests, as well as their eggs and instars, while maintaining fruit quality. Detection of a single living fruit fly at the egg, larval or mature stage will result in the immediate rejection or destruction of the whole shipment, and consequent economic loss. In addition, the quarantine treatment should be cost-effective. A mandatory cold-based quarantine treatment of 14–18 days at 1.1–2.2°C was approved by the USDA for avocado and other fruit species against the Mediterranean fruit fly *C*. *capitata* [6]. Cold-quarantine treatment has been commercially applied in citrus fruit for more than a decade, with prior heat treatment to reduce CI [40]. A combined treatment consisting of bagging in plastic and hot-water brushing was applied to pepper fruit to reduce CI during cold quarantine [41]. Recently, LTC during late-season harvest of ‘Wonderful’ pomegranate fruit opened up the possibility of cold-quarantine application [42].

Avocado cultivars Ettinger and Hass are relatively tolerant to cold and are stored commercially at 5°C [7]; however, storage below 5°C causes CI. A prior heat treatment reduced CI in ‘Sharwil’ avocado fruit during cold-quarantine treatment [24, 25]. Later research showed that LTC is more efficient than the heat treatment at reducing CI in ‘Hass’; however, a combination of LTC and heat treatment did not improve the reduction in CI [27]. We combined LTC treatment with MA [11] and MJ [18], both of which are known to reduce CI. Each therapeutic treatment alone (MA, MJ or LTC) had a significant protective effect against CI in ‘Hass’ and ‘Ettinger’. Interestingly, the combination these treatments gave a much improved protective effect against CI after 15 days of suboptimal temperature cold storage at 1°C and also after an additional 7 days of shelf storage at 20°C. This integrated therapeutic management resulted in even better fruit quality after cold-quarantine treatment than that of fruit stored commercially at 5°C. In ‘Hass’, LTC alone reduced CI severity to a satisfactory level. Moreover, both the combined treatments in ‘Ettinger’ and ‘Hass’ and the LTC treatment in ‘Hass’ significantly reduced fruit decay and increased fruit shelf life. The LTC does not required any additional cost, while both MA and JA are minimal, thus the technique should be cost-effective. These findings suggest that ‘Hass’ and ‘Ettinger’ avocado fruit can handle cold-quarantine treatment while maintaining their quality.

1-MCP is known to delay CI in avocado [36]. This study also showed that 1-MCP reduces CI and decay in ‘Hass’. However it did not have an additive effect with the LTC treatment. Nevertheless, due to its positive effect, 1-MCP should be considered in future experiments for new combinations of treatments that might reduce CI in other fruit species or avocado cultivars.

To understand the effect of combined therapeutic treatments on avocado fruit's response to suboptimal temperature storage, we monitored the relative expression of genes that are known to be induced in response to chilling. LOX, FAD and HSP are biochemical markers for postharvest chilling stress in fruit and vegetables [10, 28, 29, 43–45], and their increased expression has been found in several fruit species in response to chilling stress [10, 28, 29, 43–47]. Indeed, in our experiment, *LOX*, *FAD* and *HSP* were significantly differentially upregulated in avocado fruit stored at 1°C vs. 5°C (Fig 7).

LOX catalyzes the first step in MJ synthesis via peroxidation of α-linolenic acid, which increases during chilling stress [32, 45]. LOX also reduces membrane integrity during chilling stress. Increased LOX activity has been correlated with increased CI in loquat, cucumber, tomato and maize [28, 30, 44, 45]. Similarly, in our study, *LOX* expression was significantly induced in fruit stored at 1°C. The combined therapeutic treatments significantly reduced *LOX* expression in fruit stored at 1°C to the expression level in fruit stored at the commercial temperature (Fig 6). Thus, the combined treatments protect fruit from cold stress and alleviate CI.

Lipid peroxidation is a known indicator of CI [37, 38] and its fluorescence has been observed in avocado fruit [39]. Here we detected the luminescence of peroxidized lipids in avocado fruit stored at 1°C using IVIS. The method is non-invasive and found to be effective in detection of lipid peroxidation in whole fruit. It's reproducibility was confirmed previously in various cultivars of mango [34]. The observed increase in luminescence in chilling-stressed fruit stored at 1°C corroborated well with the increase in *LOX* expression—which catalyzes membrane lipid peroxidation and results in CI. Reduced level of luminescence in fruit stored at 1°C with combined therapeutic treatments proved that the treatments effectively minimized CI and lipid peroxidation.

HSP II 17.6 increases chilling tolerance in fruit by stabilizing the membrane and scavenging radicals [10]. FAD is induced by cold stress and converts membrane saturated fatty acids (FA) to unsaturated FA. Thus, it maintains membrane integrity and prevents CI in fruit [29]. In our study, expression of both *HSP* and *FAD* was immediately activated at suboptimal temperature storage at 1°C and started to decline on days 4 and 8, respectively. Interestingly, in the treated fruit, the expression of *HSP* and *FAD* gradually increased to a peak on day 18 (at significantly higher levels than non-treated fruit stored at 1°C or 5°C). Thus, HSP and FAD are induced by therapeutic treatments and probably protect the fruit from CI.

The mechanisms of action of LTC, MA, and MJ's protection of fruit from CI is not clear. Nevertheless, a recent review described some possible functions attributed to CI alleviation in fruit and vegetables treated with MJ, among them a decrease in LOX activity, and increase in FAD and HSP gene expression [29]. Tomato fruit pretreated with MJ showed induced HSP expression and acquired resistance against CI [46]. Loquat fruit treated with MJ showed decreased LOX activity and an increase in membrane lipid unsaturation [30]. LTC treatment induced FAD levels in loquat fruit [31]. Accordingly, in our study, the therapeutic treatments decreased *LOX* expression and lipid peroxidation and enhanced *FAD* and *HSP* expression at a later time point, thereby protecting avocado fruit from cold stress and alleviating CI.

Overall, cold-quarantined avocado fruit show severe CI, lipid peroxidation, and increased expression of *LOX*, *FAD* and *HSP* genes. LOX might play a crucial role in the induction of CI in avocado fruit. A combination of physical and chemical therapeutic treatments (MA, MJ and LTC) alleviated this CI, probably by inducing defense pathways and reducing *LOX* expression. A successful cold-quarantine method against *C*. *capitata* was developed for ‘Hass’ and ‘Ettinger’ avocado fruit, without affecting fruit quality. Moreover, treated fruit stored at low temperature (1°C) had a longer shelf life and less decay than non-treated fruit stored at the commercial storage temperature.

## Conclusions

Quarantine treatment is essential for pest disinfestation in fruit and its international trade [1]. The developed cold-quarantine treatment for avocado with prior application of a combination of therapeutic treatments will potentially enable exporting avocado fruit to quarantine-requiring countries and allow its global transport. Thus, optimally, avocado fruit could be available in markets throughout the year.

## Supporting Information

S1 Fig Fruit pulp temperature. Avocado ‘Hass’ fruit pulp temperature during suboptimal temperature storage at 1°C and further shelf storage: (A) without therapeutic treatments, (B) with the combined therapeutic treatments [modified atmosphere (MA), methyl jasmonate (MJ) and low-temperature conditioning (LTC)].
(TIFF)

S2 Fig Evaluation of internal browning in ‘Hass’ and ‘Ettinger’ after cold storage and further shelf storage. Representative pictures of ‘Hass’ after cold storage at 5°C (negative control), at 1°C without treatment (positive control) and at 1°C with combined treatments [modified atmosphere (MA), methyl jasmonate (MJ) and low-temperature conditioning (LTC)] followed by further shelf storage.
(TIFF)

S3 Fig Effect of combined therapeutic treatments on shelf-life extension. Representative pictures of ‘Ettinger’ fruit after 3 weeks of cold storage at 5°C (negative control), at 1°C without treatment (positive control) and at 1°C with combined treatments [modified atmosphere (MA), methyl jasmonate (MJ) and low-temperature conditioning (LTC)], followed by 10 days of shelf storage (SL).
(TIFF)
