# Supplementary figures and images for: Combined Treatments Reduce Chilling Injury and Maintain Fruit Quality in Avocado Fruit during Cold Quarantine
Source: PLoS One. 2015 Oct 26;10(10):e0140522. doi: 10.1371/journal.pone.0140522 (PMC4621022; doi:10.1371/journal.pone.0140522)

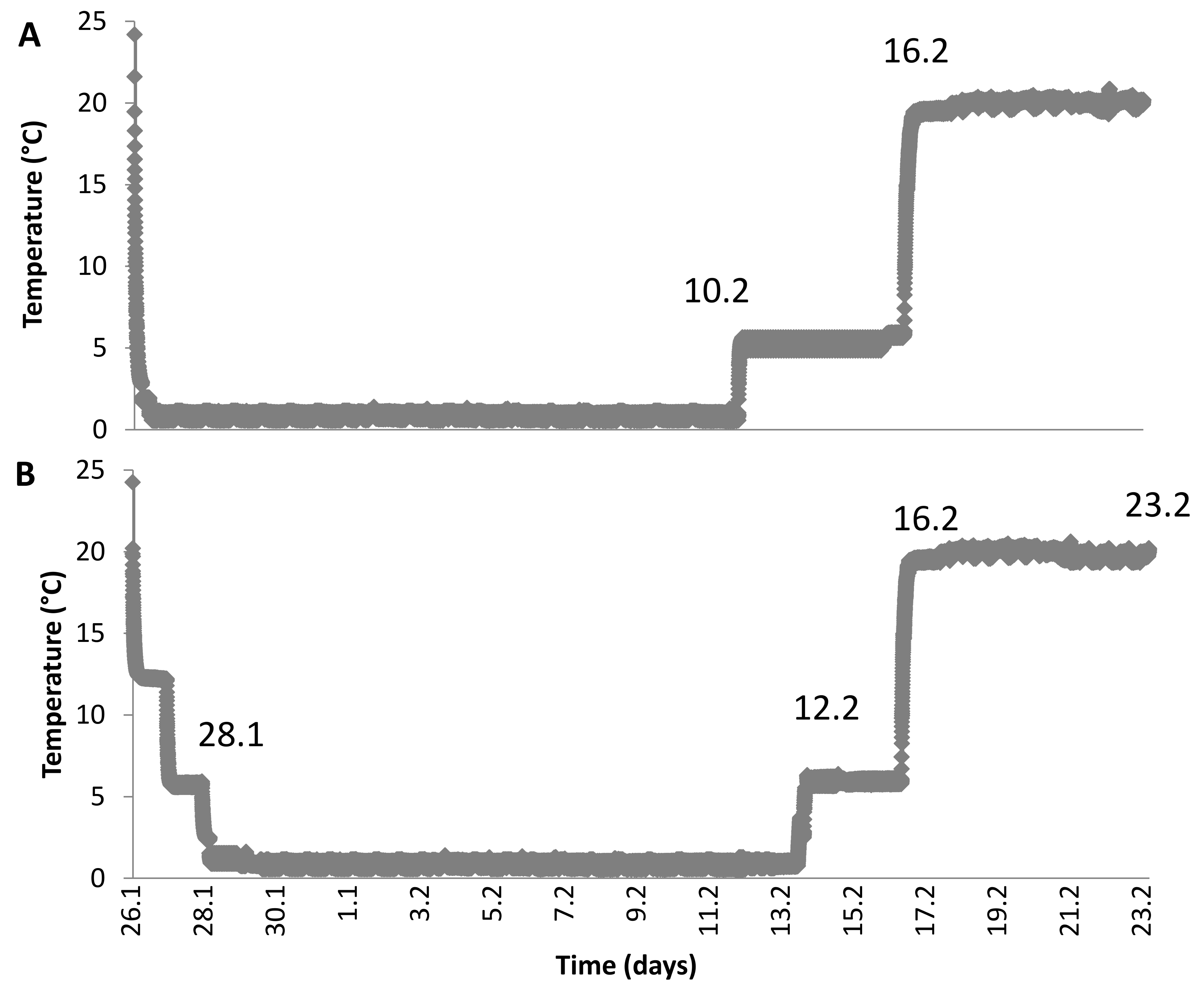

Supplement: S1 Fig — Avocado ‘Hass’ fruit pulp temperature during suboptimal temperature storage at 1°C and further shelf storage: (A) without therapeutic treatments, (B) with the combined therapeutic treatments [modified atmosphere (MA), methyl jasmonate (MJ) and low-temperature conditioning (LTC)]. (TIFF) [file pone.0140522.s001.tiff]

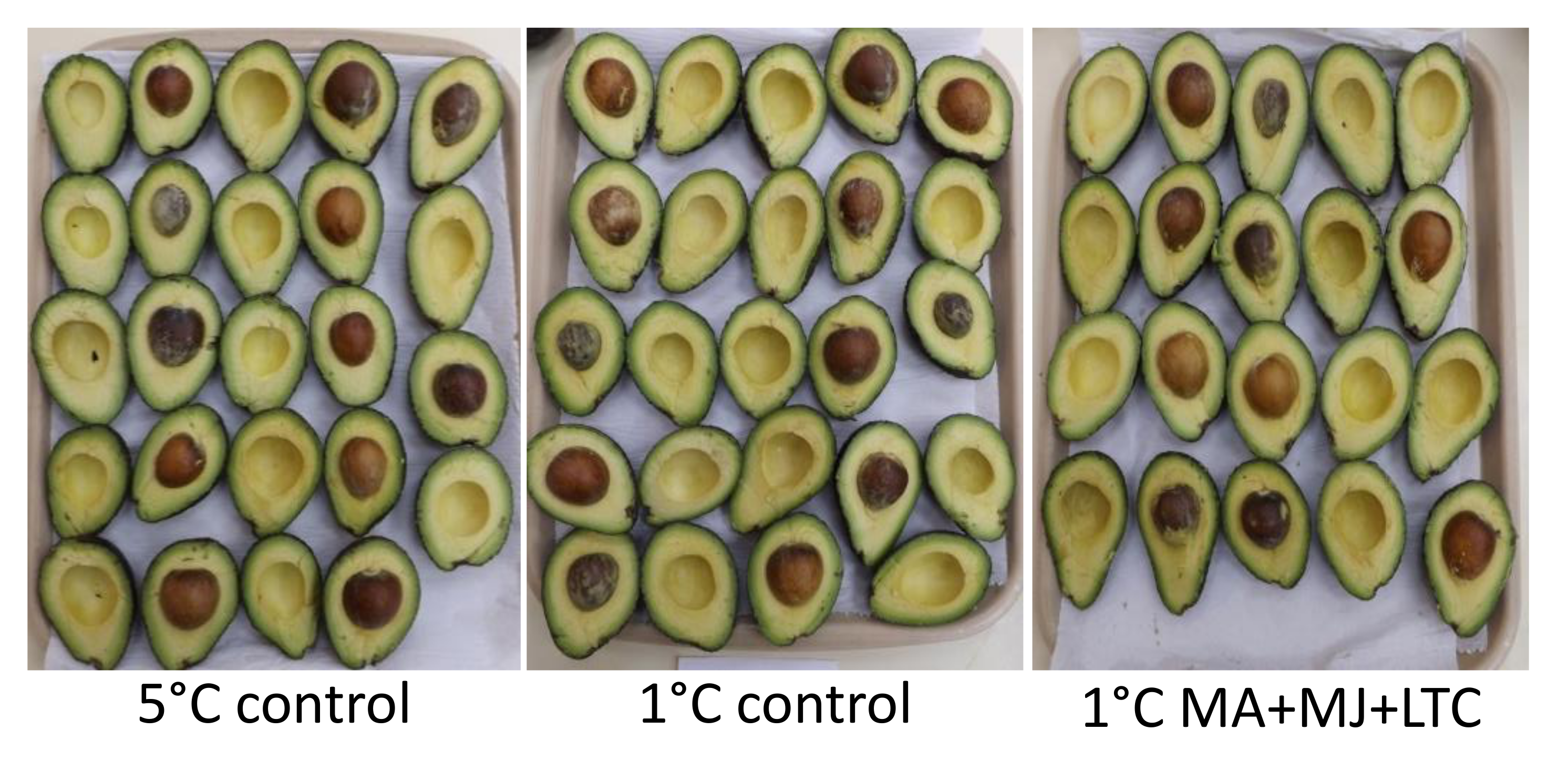

Supplement: S2 Fig — Representative pictures of ‘Hass’ after cold storage at 5°C (negative control), at 1°C without treatment (positive control) and at 1°C with combined treatments [modified atmosphere (MA), methyl jasmonate (MJ) and low-temperature conditioning (LTC)] followed by further shelf storage. (TIFF) [file pone.0140522.s002.tiff]

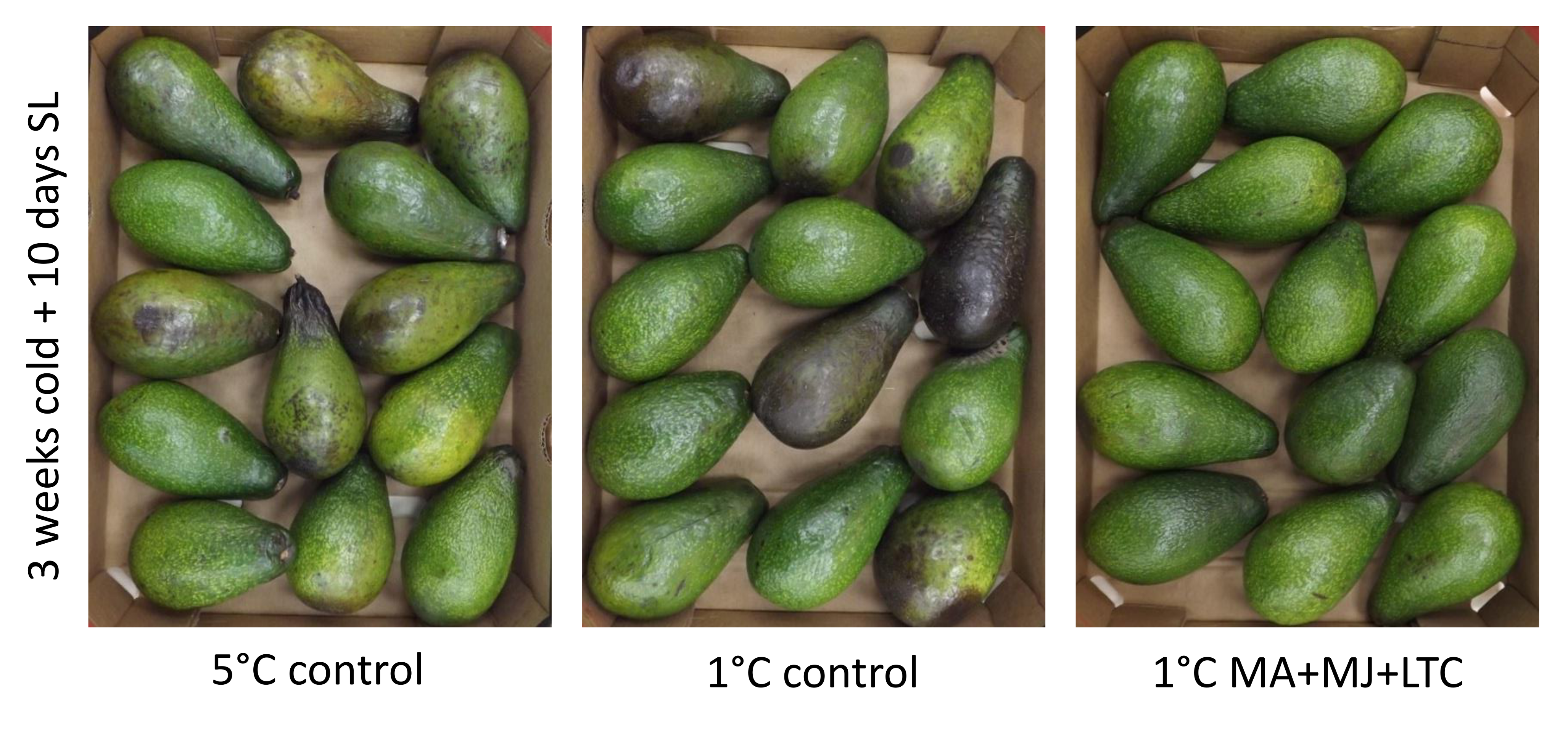

Supplement: S3 Fig — Representative pictures of ‘Ettinger’ fruit after 3 weeks of cold storage at 5°C (negative control), at 1°C without treatment (positive control) and at 1°C with combined treatments [modified atmosphere (MA), methyl jasmonate (MJ) and low-temperature conditioning (LTC)], followed by 10 days of shelf storage (SL). (TIFF) [file pone.0140522.s003.tiff]
